# Chemotherapy plus dendritic cells co-cultured with cytokine-induced killer cells versus chemotherapy alone to treat advanced non-small-cell lung cancer: A meta-analysis

**DOI:** 10.18632/oncotarget.13394

**Published:** 2016-11-16

**Authors:** Cuiling Zhou, Donglan Liu, Jie Li, Huanhuan Sun, Xiaobin Zheng, Shuncong Wang, Guobin Hong, Saradhi Mallampati, Hongliu Sun, Xiuling Zhou, Zhibin Cheng, Hongyu Zhang, Haiqing Ma

**Affiliations:** ^1^ Department of Oncology, The Fifth Affiliated Hospital of Sun Yat-sen University, Zhuhai, Guangdong 519000, China; ^2^ Department of Gastroenterology, Cancer Hospital of Jiangxi Province, Nanchang, Jiangxi 330029, China; ^3^ Department of Breast and Thyroid Surgery, The First Affiliated Hospital of Sun Yat-sen University, Guangzhou, Guangdong 510080, China; ^4^ Department of Respiratory, The Fifth Affiliated Hospital of Sun Yat-sen University, Zhuhai, Guangdong 519000, China; ^5^ Department of Radiology, The Fifth Affiliated Hospital of Sun Yat-sen University, Zhuhai, Guangdong 519000, China; ^6^ Department of Laboratory Medicine and the Center for Stem Cell and Developmental Biology, The University of Texas MD Anderson Cancer Center, Houston, TX 77030, USA; ^7^ Department of Pathology, University of Michigan, Ann Arbor, MI 48201, USA

**Keywords:** advanced non-small-cell lung cancer, overall survival, combination treatment of DC-CIKs, chemotherapy, meta-analysis

## Abstract

This study was aimed to investigate the efficacy and safety of the combination treatment of dendritic cells co-cultured with cytokine-induced killer cells and chemotherapy for patients with advanced non-small-cell lung cancer (NSCLC). Literatures were searched from the Cochrane Library Central, PubMed, Web of Science and EMBASE. The primary endpoint of interest was overall survival (OS), and secondary endpoints were disease control rate (DCR) and progression free survival (PFS). Finally 7 trials published between January 2005 and March 2016 met inclusion criteria and totally 610 patients were enrolled. The combination group showed advance in DCR (RR = 1.31, 95% CI = 1.13-1.52, *p =* 0.0004), 1-year OS (RR = 1.18, 95% CI = 1.05-1.33, *p* = 0.007), and 2-year OS (RR = 1.37, 95% CI = 1.10-1.70, *p* = 0.005), with statistical significance. The proportions of CD3^+^ T cells (*p* = 0.002), NK cells (*p* = 0.02) and NKT cells (*p* = 0.001) were significantly higher in the peripheral blood of combination group, compared with those of the control group. Moreover, adverse reactions were obviously decreased in the combination group. However, no significant difference was identified in ORR and PFS between two groups (*p* > 0.05). In conclusion, the combination therapy was safe and applicable for patients with advanced NSCLC.

## INTRODUCTION

Lung cancer has been considered a very fateful disease around the world with the 5-year survival rate of roughly 15%. Moreover, majority of patients are diagnosed as advanced cancer [1]. Approximately 80% of lung cancer cases are non-small-cell lung cancer (NSCLC) [2]. Common therapies include surgery, chemotherapy and radiotherapy, however, they fail to completely remove the tumor cells [3]. Recently, there has been growing interest in adoptive cellular immunotherapy, which is considered as the fourth method following three common therapies, for it can reconstruct the immune system, thus has an important impact on killing small lesions and bone marrow purification. Adoptive cellular immunotherapy is an art, amplifying the autologous tumor-specific effector cells *ex vivo* and re-infusing them into the host. Since conventional therapies could not completely eradicate tumor cells, the killing effect of adoptive cellular immunotherapy to tumor cells is an important supplement to conventional therapies [4, 5].

Dendritic cells (DCs) and cytokine-induced killer cells (CIKs) are two important components of adoptive cellular immunotherapy. The most potent antigen-presenting cells in the body are DCs, which promote the generation of helper and cytotoxic T cells. Therefore they are responsible for the initiation of both innate and adoptive immune responses [6, 7]. DCs play an important role in controlling immunity versus tolerance, microbial infections, autoimmune diseases and antitumor immune responses [8]. CIKs are a heterogeneous subset of T lymphocytes, showing mixed T-NK phenotypes, and can be harvested from bone marrow or peripheral blood mononuclear cell [9]. As reported by Schmidt-Wolf, CIKs play a crucial role in bone marrow purging for autologous bone marrow transplantation [10]. In addition, the reasons of increased anti-tumor activity of CIKs are mainly as follows: high proliferation rate of the CD3^+^CD56^+^ phenotype, increased efficacy with few adverse events, and non-MHC-restricted killing [11].

Co-culturing with DCs enhanced the cytotoxic activity of CIKs, since the proportion of CD3^+^CD8^+^ cells and levels of cytokines such as IL-8, IFN-γ and TNF-α significantly increased in CIKs co-cultured with DCs than in simple CIKs. CIKs co-cultured with DCs can release large number of toxic particles and inflammatory cytokines, thus inducing tumor cell apoptosis [12]. Several results showed that the combination of DCs and CIKs were more effective and indicated more promising clinical prospects than single CIKs treatment [13, 14]. DC-CIKs immunotherapy has been widely used in solid and hematopoietic tumors, such as breast cancer, renal cell carcinoma, gastric cancer, colorectal cancer and leukemia [12, 15–17]. Meanwhile, previous experiments in different degree showed DC-CIKs immunotherapy could prolong survival, relieve clinical symptoms or improve patients’ cellular immune function in NSCLC [18, 19]. A meta-analysis of advanced NSCLC showed significantly higher overall survival (OS) and disease control rate (DCR) in group with combination treatment of DC-CIKs plus chemotherapy than in chemotherapy only group, but did not report immune function [20]. To investigate the efficacy and safety of DC-CIKs immunotherapy for advanced NSCLC and thus help future clinical trials, this meta-analysis was conducted by comparing the combined application of DC-CIKs and chemotherapy with chemotherapy alone.

## RESULTS

### Search results

**Figure 1:**
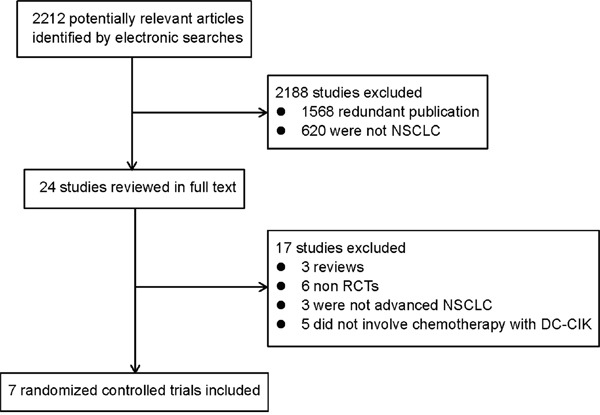
Flow diagram of the study selection process

A total of 2212 records were identified during initial literature search. After duplicate removal and abandoning the article not related to NSCLC, 24 studies were reviewed. Of these, 17 papers were excluded for the following reasons: 3 studies were review articles; 3 studies were not about advanced NSCLC; 6 studies were not randomized controlled trials (RCTs), and 5 studies did not involve chemotherapy with DC-CIKs immunotherapy. Finally, 7 trials including a total of 610 patients were recruited in the meta-analysis (Figure 1).

**Figure 2:**
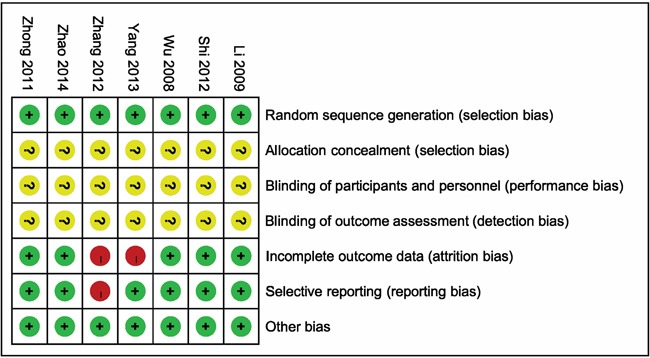
Risk of bias summary: reviewing authors’ judgments about each risk of bias item for each included study

After reviewing full text, the data of all studies were summarized in Table 1, and risk of bias summary was presented in Figure 2, by reviewing the authors’ judgments about each risk of bias item for each included study.

**Table 1:** Clinical information of the eligible trails for the meta-analysis

Study	Nation	Patients	Follow up (Year)	Gender(F/M)		Median Age(Range)		Treatment Design	
				Com	Con	Com	Con	Com	Con
Wu 2008 [21]	China	59	2	5/24	7/23	60.0(41-78)	61.0(38-74)	DP+CIK	DP
Li 2009 [22]	China	84	4	14/28	14/28	60.0(41-78)	60.5(40-80)	NP+DC/CIK	NP
Shi 2012 [35]	China	60	2	13/17	12/18	60.5(40-77)	58.5(40-76)	GP/DP+DC/CIK	GP/DP
Yang 2013 [36]	China	122	4	12/49	12/49	63.0(29-80)	63.5(28-82)	NP+DC/CIK	NP
Zhong 2011 [23]	China	28	7	8/6	7/7	50.0(39-68)	48.0(40-65)	NP+DC/CIK	NP
Zhang 2012 [37]	China	100	2	50	50	57.0(35-72)	58.0(37-68)	DP+DC/CIK	DP
Zhao 2014 [38]	China	157	3	30/49	32/46	59.6(32-87)	58.2(30-76)	GP+DC/CIK	GP

**Note:** A total of 610 patients were included in the meta-analysis; among these patients, 306 were assigned to the combination group (Com) treated with chemotherapy plus DC-CIKs/CIKs and 304 were assigned to the control group (Con) treated with chemotherapy alone.

**Abbreviations:** F, Female; M, Male; CIK, cytokine-induced killer biotherapy; DC, dendritic cells; DP, docetaxel-cisplatin chemotherapy; NP, vinorelbine-platinum chemotherapy; GP, gemcitabine-platinum chemotherapy.

### Efficacy assessment

**Figure 3:**
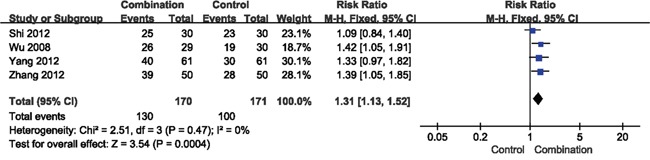
Forest plot of the comparison of disease control rate (DCR) CI, confidence interval; RR, risk ratio; Combination group, chemotherapy + DC-CIKs; Control group, chemotherapy alone.

**Figure 4:**
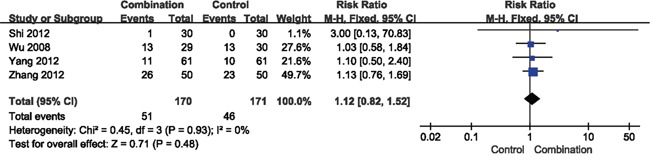
Forest plot of the comparison of overall response rate (ORR) CI, confidence interval; RR, risk ratio; Combination group, chemotherapy + DC-CIKs; Control group, chemotherapy alone.

The results of DCR showed favorable effects for the combination therapy (RR = 1.31, 95% CI = 1.13-1.52, *p* = 0.0004) (Figure 3). However, the RR of overall response rate (ORR) was 1.12 (95% CI = 0.82-1.52, *p* = 0.48), indicating that there was no significant difference between the combination and control groups (Figure 4).

### Prognosis evaluation

**Figure 5:**
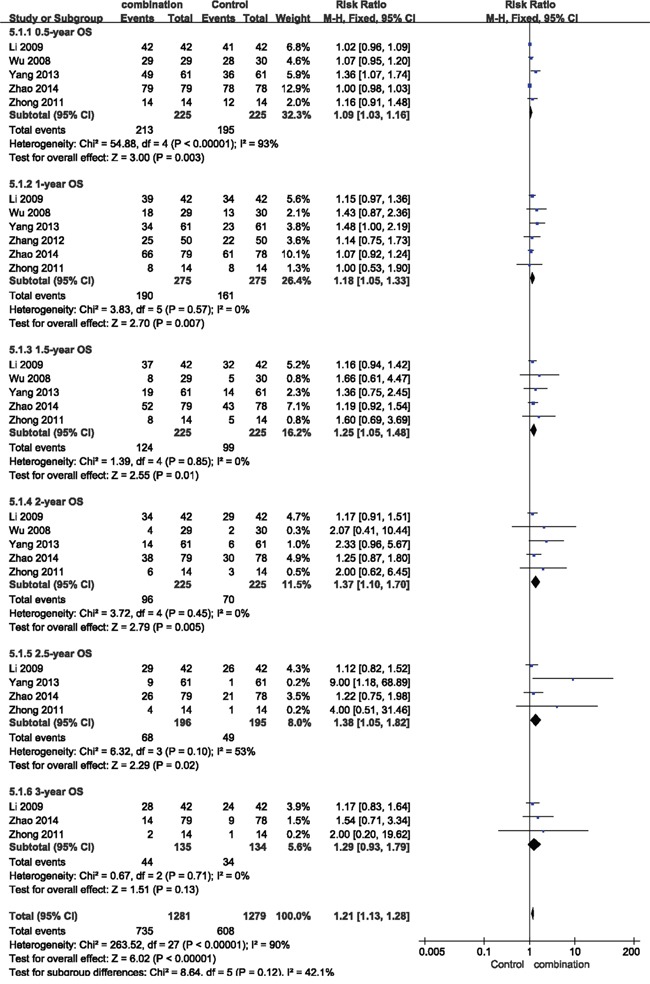
Forest plot of the comparison of overall survival (OS) CI, confidence interval; RR, risk ratio; Combination group, chemotherapy + DC-CIKs; Control group, chemotherapy alone.

The results of the pooled analysis showed that patients in combination group had a significantly improved 0.5-year OS (RR = 1.09, 95% CI = 1.03-1.16, *p* = 0.003), 1-year OS (RR = 1.18, 95% CI = 1.05-1.33, *p* = 0.007), 1.5-year OS (RR = 1.25, 95% CI = 1.05-1.48, *p* = 0.01), 2-year OS (RR = 1.37, 95% CI = 1.10-1.70, *p* = 0.005), and 2.5-year OS (RR = 1.38, 95% CI = 1.05-1.82, *p* = 0.02), whereas immunotherapy group didn't show significantly extended 3-year OS (RR = 1.29, 95% CI = 0.93-1.79, *p* = 0.13) compared with control group (Figure 5).

**Figure 6:**
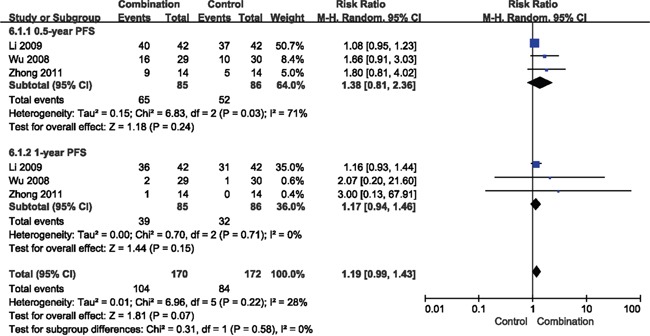
Forest plot of the comparison of progression free survival (PFS) CI, confidence interval; RR, risk ratio; Combination group, chemotherapy + DC-CIKs; Control group, chemotherapy alone.

The random-effect model was used for heterogeneity observed in the progression free survival (PFS). The patients in combination therapy group showed insignificantly prolonged 0.5-year and 1-year PFS compared with those in chemotherapy alone (RR = 1.38, 95% CI = 0.81-2.36, *p* = 0.24; RR = 1.17, 95% CI = 0.94-1.46, *p* = 0.15, respectively) (Figure 6).

### Immune responses

**Figure 7:**
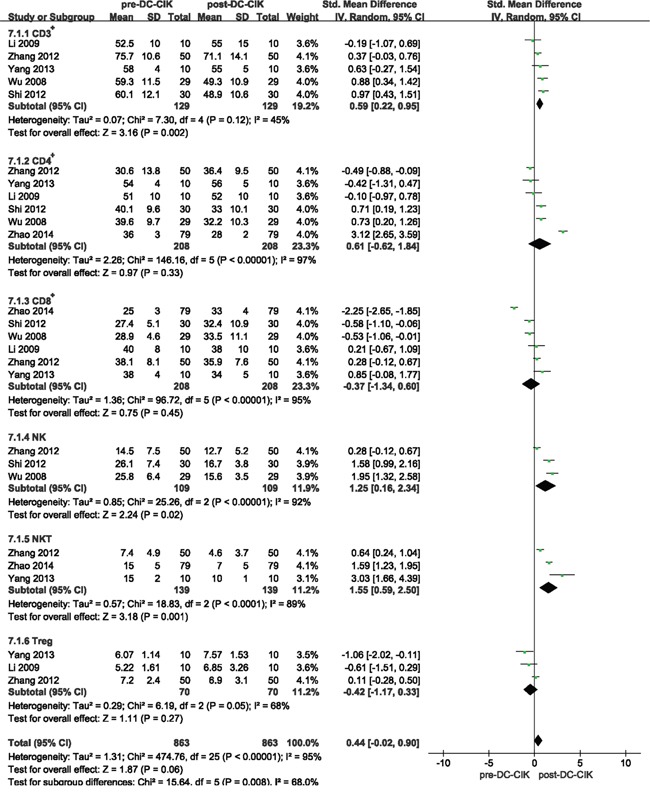
Forest plot of the immune response in pre-DC-CIK + chemotherapy and post-DC-CIK + chemotherapy CI, confidence interval; SMD, standardized mean difference; pre-DC-CIKgroup, chemotherapy alone; post-DC-CIK group, chemotherapy + DC-CIK.

When heterogeneity was tested in the T-cell subgroups, a random-effect model was selected for the subgroup analysis of immune response. The analysis showed that the ratios of CD3^+^ T cells (SMD = 0.59, 95% CI = 0.22-0.95, *p* = 0.002), NK cells (SMD = 1.25, 95% CI = 0.16-2.34, *p* = 0.02) and NKT cells (SMD = 1.55, 95% CI = 0.59-2.50, *p* = 0.001) were significantly increased in combination group than in control group. Whereas the ratios of CD4^+^ T cells, CD8^+^ T cells and regulatory T cells (Treg) showed no statistical improvement after DC-CIK treatment (SMD = 0.61, 95% CI = -0.62-1.84, *p* = 0.33; SMD = -0.37, 95% CI = -1.34-0.60, *p* = 0.45; SMD = -0.42, 95% CI = -1.17-0.33, *p* = 0.27, respectively) (Figure 7).

### Toxicity and adverse reactions

**Figure 8:**
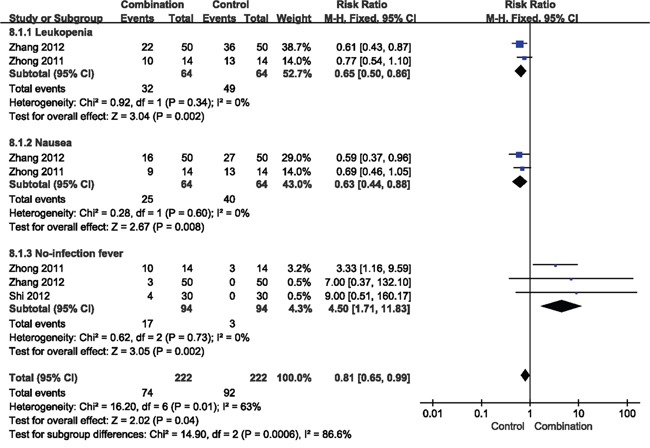
Forest plot of the comparison of the toxicity CI, confidence interval; RR, risk ratio; Combination group, chemotherapy + DC-CIKs; Control group, chemotherapy alone.

Patients in control group showed several side effects, including leucopenia, nausea and no-infection fever, which mostly also occurred in the combination group. Without significant heterogeneity, we chose a fixed-effect model to analyze side effects. On the whole, the incidence was 0.65 for leucopenia (RR = 0.65, 95% CI = 0.50-0.86, *p* = 0.002) and 0.44 for nausea (RR = 0.44, 95% CI = 0.44-0.88, *p* = 0.008). Although no-infection fever (RR = 4.5, 95% CI = 1.71-11.83, *p* = 0.002) was increased in the combination group, it was moderated by itself within 24 hours (Figure 8).

### Sensitivity analysis

Because one study did not involve DCs immunotherapy [21]. We need to perform sensitivity analyses to test the stability of our data. We observed that the overall results were still reliable when each parameter was excluded or included in sequence.

## DISCUSSION

Our meta-analysis revealed that the combination treatment of DC-CIKs with chemotherapy in advanced NSCLC could prolong OS rate and enhance DCR, but it failed to improve PFS and ORR.

Consistent with our results, Li *et al*. reported that 2-year OS in combination group was significantly higher than that of control group, although 2-year PFS between two groups showed no significant difference [22]. In our analysis, the PFS difference between combination group and control group didn't reach statistical significance. However, another two studies showed that combination group had a longer PFS compared with chemotherapy group [21, 23], indicating inconsistency regarding the difference of PFS between two groups. Reasons for this different result may be attributable to the lack of studies reporting PFS and shorter follow-up time. Importantly, the consensus is that DC-CIKs have noticeable impacts on OS and DCR in patients with NSCLC.

In our analysis, all patients in combination group received chemotherapy followed by DC-CIKs treatment. Chemotherapy as a lymphodepletion regimen before adoptive T cell transfer has been shown to substantially improve survival and anti-tumor responses of the transferred cells [24]. This could, at least partially, explain the cooperative effect of DC-CIKs treatment to chemotherapy in advanced NSCLC. In our analysis, all patients in combination group received autologous CIKs. Actually allogeneic CIKs have been reported to be administrated in lymphoma/leukemia patients who relapsed after allo-HSCT [25], and donor-derived CIK cells were well-tolerated and did not lead to more acute GVHD, suggesting that allogeneic CIKs may be applicable in the combination treatment with chemotherapy in advanced NSCLC but need more evidence in the future.

The international registry on CIK cells had reported a good response rate and significantly increased OS, accompanied by an improved quality of life and minor side effects of CIKs treatment [26]. Moreover, it's worth noting that many researchers have reported longer survival and enhanced DCR due to combined application of DC-CIKs and chemotherapy in different solid cancers, such as breast cancer, colon cancer, advanced gastric cancer and advanced renal cancer [27–29]. It further indicated the role of DC-CIKs as an effective component in the comprehensive treatment of solid cancer.

The side effects of combination group were slighter compared with those of chemotherapy alone group. In the present study, we observed higher percentages of CD3^+^ T lymphocytes, NK cells and NKT cells in combination group, indicating that autologous DC-CIKs enhance immune activity. But, no significant difference was shown in percentages of CD4^+^, CD8^+^, CD56^+^ and regulatory T cells between pre-DC-CIKs treatment and post-DC-CIKs treatment. There are some reasons accountable for these results. Firstly, the non-unified dosage regimens of DC-CIKs transfusion may give rise to various clinical outcomes including different immune responses. In addition, there was unclear testing time of subsets of lymphocyte. Therefore, a standard dosage and treatment regimens of DC-CIKs transfusion is imperative for the future.

To date, the efficacy and safety of the combination treatment of DC-CIKs with chemotherapy had been observed in advanced NSCLC patients. Several studies showed similar results to ours, however we enrolled more comprehensive trials to achieve higher statistical reliability, especially in the analyses of immunity after DC-CIKs immunotherapy [20, 30]. Besides, our literature search strategy guaranteed full coverage of related publications. Finally, the values of RR and SMD demonstrated that our results were similar to the results of all enrolled studies, suggesting the validity of our results. To sum up, our study confirmed that DC-CIKs plus chemotherapy was a safe and effective treatment for patients with advanced NSCLC.

There are some limitations in our study. Firstly, the follow-up time is not long. Secondly, our analyzed data were partly extracted from published papers rather than the original patient records, resulting in bias of the analytical results. Thirdly, our study did not enroll vast clinical cases. Due to the above limitations in our analysis, further studies are needed to verify its safety and efficacy.

Our meta-analysis demonstrated that the combination therapy was safe and applicable for patients with advanced NSCLC. It is suitable for patients with low immunity after conventional treatments via providing a feasible option in preventing from tumor recurrence, prolonging survival time and improving life quality. The combination therapy demonstrates significant superiority in terms of clinical responses, treatment efficacy, immune responses and side effects compared with the chemotherapy alone. This study may aid in paving the way for the combination therapy in other malignancies, and help promote the development of adoptive cellular therapy.

## MATERIALS AND METHODS

### Literature search

The meta-analysis data were conducted by PRISMA statement guidelines [31]. We searched literatures published between January 2005 and March 2016 from the Cochrane Library Central, PubMed, EMBASE and Web of Science. Involved articles were reported in English and studied about humans. Some searched key words included “dendritic cells and cytokine-induced killer cells” or “DC-CIK immunotherapy” or “cytokine-induced killer cells” or “dendritic cells”, “non-small-cell lung cancer” or “lung cancer” or “NSCLC”, and “chemotherapy”, and “clinical trial”, and “humans”.

### Study selection and quality assessment

The eligible RCTs were entered into our study. In the control group, patients with advanced NSCLC received chemotherapy alone, whereas patients in the combination group were treated with DC-CIKs immune therapy plus chemotherapy.

The study inclusion criteria were as follows: (1) studies were concerned with advanced NSCLC; (2) studies should be RCTs; (3) all the trials had not been mixed with other treatments in either group.

The followings were exclusion criteria: (1) original studies only assessed either the result of DC-CIKs + chemotherapy group or the result of chemotherapy alone group; (2) studies did not involve advanced NSCLC; (3) review articles, letters and case reports were not enrolled.

According to the Cochrane handbook, we assessed the quality of the included studies [32].

### Data extraction

Two reviewers (Cuiling Zhou and Huanhuan Sun) independently searched potentially relevant articles by the way of scanning titles and available abstracts. Disagreements were discussed with the third reviewer (Shuncong Wang) after going back to the original articles. All involved studies were summarized as follows: the first author's last name; year of article publication; the place of the performed study; number and characteristics of patients between the combination group and control group.

### Curative effects

The primary endpoint was OS, which was defined as the time from the initiation treatment to death or the date which patients were last known to be alive. The secondary endpoints were PFS and DCR. PFS was meant the interval between the time of starting treatment and the time of disease progression or death. DCR was the sum of stable disease, partial response and complete response, and ORR was the sum of partial response and complete response, according to the World Health Organization and International Union Against Cancer Criteria [33]. Then we evaluated the toxicity and subsets of the T-lymphocyte in the peripheral blood in the present study. Toxicity was graded in line with the National Cancer Institute Common Toxicity Criteria [34].

### Statistical analysis

The analysis was carried out with the help of Review Manager Version 5.3 (Cochrane Collaboration). *p* < 0.05 shows the existence of significant difference. Risk ratio (RR) and standard mean difference (SMD) were used as the fundamental measurements of efficacy for dichotomous data and continuous variables, respectively. A 95% confidence interval (CI) was reported as we assessed. Because of the heterogeneity of involved studies, we appropriately chose a fixed-effect method or a random-effect method. The *I^2^* statistic and *p* value were used to test statistical heterogeneity of studies, with a predefined significance threshold of *I^2^* < 50% or *p* > 0.1. Seven trials were finally included in our analysis [21–23, 35–38]. Because one trial (Wu *et al*.[21]) didn't apply DC immunotherapy in combination group, sensitivity analysis was necessary.
